# Dental Anxiety and Influencing Factors in Adults

**DOI:** 10.3390/healthcare10122352

**Published:** 2022-11-23

**Authors:** Muhammad Usman Muneer, Fahad Ismail, Nadia Munir, Asma Shakoor, Gotam Das, Abdul Razzaq Ahmed, Muhammad Adeel Ahmed

**Affiliations:** 1 Department of Prosthodontics, Avicenna Dental College, Lahore 53100, Pakistan; 2 Department of Dental Materials, Avicenna Dental College, Lahore 53100, Pakistan; 3 Department of Community and Preventive Dentistry, Institute of Dentistry, CMH-Lahore Medical College, National University of Medical Sciences, Lahore 54000, Pakistan; 4 Department of Prosthodontics, College of Dentistry, King Khalid University, Abha 61421, Saudi Arabia; 5 Department of Restorative Dentistry and Endodontics, College of Dentistry, King Faisal University, Al Ahsa 31982, Saudi Arabia

**Keywords:** dental anxiety, dental treatment, avoidance

## Abstract

Dental anxiety is one of the most common conditions present amongst the masses globally. It is this fear that makes individuals avoid seeking dental treatment which results in a deteriorated oral health-related quality of life (OHRQoL). Discrepancies exist in the prevalence of dental anxiety based on gender, education levels, level of deprivation of a society and its socioeconomic status. In this study, a sample size of 522 respondents was collected. Kuppuswamy’s socioeconomic status scale and modified dental anxiety scales were used to collect the necessary data. These data were analyzed by cross tabbing and chi-square test of significance was applied to assess the association between dental anxiety and other factors. Female gender was significantly associated with dental anxiety with *p*-value = 0.03. Higher education levels and dental anxiety also displayed significant associations with each other, with a *p*-value of 0.048. Seventy-six percent of the individuals of lower socioeconomic status were prone to be more dentally anxious. Dental anxiety was more significant in individuals with higher levels of education in our study. Respondents who were part of a lower socioeconomic class were also more prone to being anxious while receiving dental treatment. Knowing the factors that cause dental anxiety can help dentists effectively manage and treat their patients.

## 1. Introduction

Dental Anxiety is the fear of going to see a dentist and obtaining dental treatment. Sometimes this anxiety can border on dental phobia, which acts as a significant impediment to patients seeking oral healthcare [1,2,3,4], and thus affects their oral health, and, subsequently, their quality of life [1,3,5,6]. Lack of timely attention to an individual’s teeth can cause issues such as carious teeth, periodontitis, and, consequently, the loss of natural teeth [6,7]. Dental Anxiety can be caused by a traumatic dental experience, feeling of shame, loss of control in a dental chair, distrust of a dentist, or it can be a perceived fear based on anecdotal incidents [8,9,10,11]. Dental phobia is one of the most commonly seen phobias in the world, despite the raised awareness and focus on improving the doctor–patient rapport [1].

Dental anxiety and phobia are seen in a vast number of people and understanding those who suffer from these phenomena can only help dentists manage their patients more effectively [2,12,13]. Dentists find working with a dentally anxious patient stressful and it triggers their anxiety, which can result in compromised treatment for the patient. It can also result in the dentist victim blaming the patient, which further reinforces the patient’s anxiety. [5,7]. Dental anxiety may allow, even with some difficulties, some treatments. Simple treatments, such as direct restorations in anterior [14,15] or posterior teeth [16], dental endodontic retreatments [17], or orthodontics [18] do not require dental anesthesia. Anxiety is therefore very often manageable in this type of case. When the complexity of the treatment increases, and when local anesthesia is required, other types of treatments, such as prosthetics [19] or implantology [20], may be unlikely to be performed correctly. In these cases, anxiety may turn into dental phobia and affect the treatment’s outcome.

Factors such as age, gender, and education levels play an important role in affecting dental anxiety in people. Older individuals are generally less fearful of getting dental treatment than their younger counterparts [3]. Females are more prone to report having dental anxiety and dental phobia [3]. Women are generally more expressive about experiencing pain, despite their pain threshold [5,21,22]. Men, on the other hand, hide their emotions due to cultural and social norms and hence may not report feeling anxious about dental treatment readily [23]. Women were also reported to have more dental anxiety while anticipating the treatment [11].

It is usually seen that people with higher education levels experience less dental anxiety because their understanding of the treatment is higher compared to someone who has less education [3,13,22,24]. On the flipside, ignorance is bliss, and sometimes individuals who had less awareness regarding dental treatment modalities were less inclined to be anxious about the treatment itself [22].

Finances also play a role in propagating dental anxiety [25]. A lot of times patients suffering from dental anxiety are more conscious about the payment of a treatment modality that is considered universally expensive [5,21]. Hence, people of a higher socio-economic status would be less anxious or fearful of getting dental treatment [3,6,12].

Our study aims to identify the factors that increase the dental anxiety of patients in a clinical setting in Lahore, Pakistan. This will help clinicians manage their patients effectively and create a rapport with patients to alleviate their stresses by understanding what could be triggering their anxiety and targeting that specifically.

## 2. Methods

This was a cross-sectional, quantitative survey-based study that was administered on walk-in patients in the Dental OPD of Avicenna Dental College, Lahore Pakistan. The study was conducted from December 2021 to April 2022. Permission from the research board of the college was acquired to conduct this study. The sample size was calculated using Cochrane’s Analysis, with a 5% margin error and a 95% confidence level of385.After piloting the study on 30 respondents, which incorporated an Urdu version of the Modified Dental Anxiety Scale, the data were collected by two different dentists in the college who were trained in administering the questionnaire via a hand-delivered and collected paper-based survey. A total of 522 responses were collected and included to obtain optimal results.

Individuals under the age of 18 were excluded, along with respondents who had past traumatic dental history and patients on any anti-anxiety or anti-depression medications. Demographics such as age, gender, and marital status, along with medical health history, were recorded. Validated scales, such as the Modified Kuppuswamy Status Scale and Modified Dental Anxiety Scale (MDAS), were also administered to obtain the appropriate data.

### 2.1. Kuppuswamy Status Scale

This is the most widely used scale to measure the socioeconomic status of urban families in South Asia, taking three parameters into account, namely education, occupation, and income of the individual.

Total Score Socioeconomic Status

26–29 Upper Class

16–25 Upper Middle

11–15 Lower Middle

5–10 Upper Lower

<5 Lower Class

### 2.2. Modified Dental Anxiety Scale (MDAS)

This is most commonly used scale to measure dental anxiety in individuals. The scale has been translated and validated in several languages. An Urdu version of the scale was used along with the English version in the sample. It considers 5 parameters; receiving treatment the next day, sitting in the waiting room, having a tooth drilled, receiving scaling and polishing, and receiving an anesthetic via injection.

<10—No or Low Anxiety

11–18—Moderate Dental Anxiety

>19—Severe Dental Anxiety/Dental Phobia

### 2.3. Data Evaluation

Results were analysed using SPSS (V. 23 IBM Corporation, Armonk, NY, USA, 2016). The data wereevaluated by cross tabbing MDAS scores with gender, education levels, and the Kuppuswamy socioeconomic status scale. The chi-square test was applied to evaluate the significant associations between MDAS and the other variables.

## 3. Results

Five hundred and twenty-two respondents participated in the study and the demographic details of the respondents, along with their medical status, are given in Table 1.

It was seen that 75% of the female respondents had an MDAS score >10 which denotes dental anxiety, with 20% of the female respondents reporting having dental phobia as compared to the 11% dental phobic male respondents, with 67% of the male respondents having an MDAS score >10 overall as seen in Table 2. The association between increased dental phobia and the female gender was significant (*p*-value = 0.038).

An MDAS score >10 was seen in respondents who had a professional (67%) and graduate education (77%) level, implying dental anxiety in these groups. Only 19% of the professional level and 15% of the graduate level respondents reported having a dental phobia. On the other hand, respondents with the education level of middle school (58%), primary school (66%) and lack of schooling (70%) had MDAS scores >10, with few of those respondents reporting having a dental phobia, as seen in Table 3. The association between the higher rate of education and increased dental anxiety was significant (*p*-value = 0.048).

When comparing dental anxiety to the Kuppuswamy socioeconomic status of the respondents, it was seen that the respondents who reported being a part of the upper class and upper middle class had an MDAS score >10, i.e., 50% and 70%, respectively, implying dental anxiety in these groups, with only 50% of the upper class and 16% of the upper middle-class respondents reported having the dental phobia. Of the respondents in the upper lower and lower class, 18% and 90% reported having dental anxiety, respectively, and 18% of the upper lower and 10% of the lower class reported having dental phobia (Table 4). The chi-square value was insignificant (*p*-value = 0.06).

## 4. Discussion

Dental Anxiety, or in its severe form, dental phobia, acts as a massive barrier to seeking out dental care. Lack of proper dental care results in a poor quality of oral health (OHRQoL) and ultimately a deteriorated quality of life. Dentists need to understand the factors that aggravate feelings of dental anxiety in their patients in order to treat them effectively and tactfully. It is also necessary to raise awareness about these factors because dentists themselves can perpetuate these circumstances, which are detrimental to the overall treatment of patients.

The modified dental anxiety scale is one of the most commonly used scales to measure dental anxiety. It is a tool that has been translated and validated in several languages, including Urdu. Similarly, Kuppuswamy’s socioeconomic status scale has been used widely in research in the South Asian subcontinent and was a tool that we used in this study as well [26]. The study focuses on the factors such as gender, educational level, and socioeconomic status, which may or may not aggravate feelings of dental anxiety in the masses. 

The present study sheds light on the association between gender and dental anxiety. Females were more prone to suffering from dental anxiety and dental phobia than their male counterparts, with 75% of female respondents reporting being dentally anxious. This significant association was echoed in many other kinds of research [12,13,21,27,28]. Deogade et al. and Waseem et al. attributed it to women’s readiness to express their anxiety more often than their male counterparts who may not be as open about their fears because of social stigmas [3,21].

Education also plays an important role in dental anxiety. It was seen in several studies that low education levels equate to increased dental anxiety, which could be because of the fear of the unknown [2,3,22,24,28]. Interestingly enough, the present study showed a slightly different result; people with higher education levels were significantly more anxious about getting dental treatment, and this was a trend seen in only a few other studies [23,29]. This trend can be attributed to greater awareness of the treatment modalities that end up instilling more fear in the respondents, rather than alleviating their concerns. Awareness being raised by the dentists themselves, as opposed to anecdotal information, can, however, help patients who already have a vague understanding of what a dental procedure might entail. 

It was seen in the present study that people of a lower socioeconomic status were more prone to have anxiety regarding receiving dental treatment. This trend was consistent with numerous other studies as well, all of which concluded that a poor socioeconomic status created more dental anxiety [3,5,6,12]. Most of the time it can be because of the cost of the dental treatment [5,21], which is deemed to be a more expensive treatment modality. It was clear that communities that were more deprived of necessities were more prone to have dental anxiety as opposed to communities that were not deprived [1,6,30].

This study corroborates the trends seen in studies performed in the past in a Lahore, Pakistan sample. Dental anxiety is indeed closely linked to factors such as gender, age, socioeconomic status, and education levels of individuals. These factors are related to exposure of the individuals to dentists and their treatment, a greater understanding of the morbidities of life, and the ability to afford dental treatment. Understanding these factors helps dentists raise appropriate awareness and allows patients to gain the correct understanding of dental treatments which can help alleviate their concerns adequately.

One of the major limitations of the study was using patients presenting to the hospital which prevented the gathering of more diverse data in terms of socioeconomic status and education levels. Secondly, the questionnaires were administered by the researchers which can create bias by either over-estimating or under-estimating responses along with directed elaboration of cues to help respondents understand the questions.

## 5. Conclusions

Dental Anxiety is consistently present in the masses of Lahore, Pakistan despite efforts to raise dental awareness and the proactive approaches by dentists to bridge the gap between dentistry and the patients who need it.

The following trends were concluded from our study:

- Women were more inclined to admit to being dentally anxious in the sample and hence were perceived to be more fearful of getting dental treatment.
- People of a lower socioeconomic status were reported to have more dental anxiety.
- Respondents with higher education levels were more apprehensive about seeking dental care, whereas the people with a lower education level were less anxious about it.

More widespread research into the factors affecting dental anxiety and its ill effects in society needs to be conducted to ensure well-balanced data that represents a sample of the entire population. Furthermore, there is a call to perform research that deals with factors that aggravate dental anxiety and how to control them to make dental treatment more accessible for patients.

## Figures and Tables

**Table 1:** Demographics of Respondents.

Sr. No	Demographics (N)	
1	GENDER	
	Male	276
	Female	246
2	AGE	
	20–45	338
	46–55	98
	56–65	60
	>65	26
3	MARITAL STATUS	
	Single	196
	Married	317
	Widowed	9
4	MEDICAL STATUS	
	Medically Fit	378
	Medically Compromised	144

**Table 2:** Dental Anxiety scores according to Gender.

Sr. No	MDAS Score	Gender N (%)	
		Male (274)	Female (246)
1	1–9	86 (31%)	60 (24%)
2	10–18	156 (56%)	136 (55%)
3	>19	32 (11%)	50 (20%)

**Table 3:** Dental Anxiety Scores according to Education Level.

Sr. No	MDAS Score	Education Level						
		Professional (154)	Graduate (186)	Intermediate (42)	High School (32)	Middle School (44)	Primary School (24)	Illiterate (40)
1	1–9	50 (32%)	42 (22%)	6 (14%)	10 (31%)	18 (40%)	8 (33.3%)	12 (30%)
2	10–18	74 (48%)	116 (62%)	26 (57%)	20 (62%)	24 (54%)	12 (50%)	22 (55%)
3	>19	30 (19%)	28 (15%)	10 (23%)	2 (6%)	2 (4%)	4 (16%)	6 (15%)

**Table 4:** Dental Anxiety scores according to Kuppuswamy Status.

Sr. No	MDAS Score	Kuppuswamy Socioeconomic Scale				
		26–29 (4)	16–25 (224)	11–15 (100)	5–10 (174)	>5 (20)
1	1–9	2 (50%)	66 (29%)	32 (32%)	44 (25%)	2 (10%)
2	10–18	0	122 (54%)	58 (58%)	98 (56%)	16 (80%)
3	>19	2 (50%)	36 (16%)	10 (10%)	32 (18%)	2 (10%)

## Data Availability

Not applicable.
